# Utilization of evidence-based guidelines for prevention of obesity and hypercholesterolemia among primary healthcare physicians in southwest of Saudi Arabia

**DOI:** 10.1097/MD.0000000000028152

**Published:** 2021-12-10

**Authors:** Ibrahim M. Gosadi

**Affiliations:** Department of Family and Community Medicine, Faculty of Medicine, Jazan University, Jazan, Saudi Arabia.

**Keywords:** guidelines, hypercholesterolemia, Jazan, obesity, prevention, primary healthcare, Saudi Arabia

## Abstract

To evaluate knowledge and use of evidence-based guidelines for prevention of obesity and hypercholesterolemia among primary healthcare physicians in Jazan, Saudi Arabia.

This cross-sectional study targeted 170 primary healthcare centres (PHCs) in Jazan. Measurement of study's variables was completed during personal interviews. The content of the study instrument was based on The Saudi Guidelines on the Prevention and Management of Obesity and The Cholesterol Management Guide for Healthcare Practitioners.

A total of 234 physicians participated in this study. The age of the physicians varied between 25 and 65 years and 51.7% of them were females. Knowledge of the physicians about the eat-well plate recommendations was very low, with none of the physicians reporting the recommended daily portions of fruits and vegetables. Less than 20% of the physicians knew the cutoff points for considering central obesity among male and female individuals. Only 21% of the physicians reported adherence concerning screening for obesity and 42% reported adherence to the guidelines concerning screening for hypercholesterolemia. Only 9% of the physicians were adherent to the guidelines concerning reducing the risk of obesity and only 13% reported adherence to the guidelines related to the reduction of hypercholesterolemia risk.

The current investigation detected a low level of knowledge and adherence concerning the evidence-based practice related to prevention of the obesity and hypercholesterolemia and therefore limited role of PHC physicians in the prevention of obesity or hypercholesterolemia.

## 1 Introduction

Overweight and obesity are preventable risk factors of several chronic noncommunicable conditions including raised blood sugar, raised blood pressure and cancers.^[1]^ Additionally, the excessive accumulation of fat can lead to elevated levels of triglycerides and low density lipoprotein–cholesterol.^[2–4]^ According to the World Health Organisation, 1.9 billion individuals were classified as overweight in 2016,^[5]^ and according to the Saudi Health Interview Survey, 28.7% of adult Saudis were classified as obese, with a body mass index (BMI) exceeding 30. Furthermore, the prevalence of hypercholesterolemia among Saudis was reported to reach 9.5% among Saudi males and 7.3% among Saudi females, with the prevalence being higher among Saudis above 65 years old.^[6]^

The high prevalence of overweight and obesity in Saudi Arabia can be explained by the obesogenic environment in the country. The obesogenic environment was augmented by the westernised lifestyle and barriers leading to reduced levels of physical activity.^[7]^ Physical activity barriers were reported to be more prevalent among females in Saudi Arabia, leading to a higher prevalence of overweight and obesity among females living in the country.^[8]^ Furthermore, studies measuring levels of overweight and obesity among Saudi children and adolescents suggest a further increase of obesity rates among Saudis in the coming years.^[9–11]^

Clinical guidelines have been developed to provide an evidence-based approach toward the management and prevention of obesity and hypercholesterolemia. The Saudi Guidelines on the Prevention and Management of Obesity were developed by the Obesity Control Program, Saudi Ministry of Health. The guidelines were aiming to assist practicing clinicians in Saudi Arabia in prevention and management of obesity utilising an evidence-based approach.^[12]^ Obesity prevention components involved multiple areas including risk assessment, and guidelines for healthy eating and engagement to a higher level of physical activity and the clinical criteria for the initiation of pharmacotherapy and referral for bariatric surgery.

The Cholesterol Management Guide for Healthcare Practitioners was developed by the American Heart Association, and provides guidance concerning the primary prevention of hypercholesterolemia. The guide involves methods of assessing risk of developing atherosclerotic cardiovascular disease. Depending on atherosclerotic cardiovascular disease risk, the recommended risk modifiers can be related to modification of lifestyle or initiation of pharmacotherapy to reduce low density lipoprotein–cholesterol such as Statin.^[13]^

Risk factors pertaining to obesity and hypercholesterolemia can be prevented. Furthermore, the early detection of individuals suffering from obesity and hypercholesterolemia can aid in the better management and modification of the incidence of related complications. The contribution of primary healthcare physicians (PHCs) in the prevention of obesity and hypercholesterolemia via primary prevention and screening in Saudi Arabia is currently not clear. This study aims to measure knowledge of PHC physicians in Jazan region about clinical guidelines pertaining to the prevention of obesity and hypercholesterolemia. Furthermore, adherence of the physicians in Jazan region concerning screening for obesity and hypercholesterolemia and the provision of lifestyle modifications for prevention and control of the diseases were similarly assessed.

## 2 Methodology

### 2.1 Study context

This was a cross-sectional study performed as part of a project to assess the utilisation of preventive services for the prevention and control of noncommunicable diseases including obesity and hypercholesterolemia in Jazan region, Saudi Arabia. This project involved PHC physicians working at 170 PHCs in Jazan region. Recruitment took place between October 2019 and January 2020 after securing ethical approval of the Jazan University Research Ethics Board (approval number REC40/3-090).

### 2.2 Data collection tool

A questionnaire was developed to measure the knowledge and adherence of physicians concerning the primary and secondary prevention of obesity and hypercholesterolemia. In addition to the measurement of physician demographics, the contents of the questionnaire were retrieved from The Saudi Guidelines on the Prevention and Management of Obesity^[12]^ and The Cholesterol Management Guide for Healthcare Practitioners.^[13]^ Questionnaire items measuring knowledge and adherence were closed-ended and open-ended questions. The questionnaire was reviewed by a panel of experts including consultants in family medicine, preventive medicine and epidemiology and was piloted on 10 physicians to assess the clarity of the questions.

### 2.3 Data collection process and analysis

Data collection was accomplished by interviews performed via trained medical students. PHCs physicians were identified upon attending targeted centres. Aims and process of data collection was explained to the approached physicians where the participation was voluntary. Consent to participate was obtained from approached physicians before commencement of interviews. No physicians’ identifiable data were collected during interviews. Data acquired from open-ended questions were analysed via a single investigator to recognise similar responses and establish the coding of answers.

Data was analysed via the statistical package for the social sciences (IBM SPSS Statistics version 25). Binary and categorical variables were summarised via frequencies and proportions. Similarly, continuous variables were summarised by means and standard deviations when data were normally distributed and by the median, interquartile range, and minimum and maximum values when non-normally distributed. The total score of knowledge was calculated by summing the number of correct answers. The level of knowledge was dichotomised into higher levels of knowledge and lower levels of knowledge using the median as a cutoff point. Similarly, level of adherence was dichotomised to higher adherence and lower adherence depending on reported adherence concerning screening and provision of interventions for patients’ lifestyle modifications. Odds ratios were calculated to assess possible associations between the level of knowledge or adherence with physician demographics. A *P* value of <.05 was considered statistically significant for the applied statistical tests.

## 3 Results

### 3.1 Participants characteristics

The number of physicians who agreed to participate in this investigation was 234; their demographic information are displayed in Table 1. The mean age of the physicians was 38 years and the proportion of male and female doctors was similar. The most frequently reported nationality was Sudanese. The majority of physicians were holders of a Bachelor Degree. The median number of years of experience was 10 years. On average, 40 patients were seen by the participating physicians on a daily basis.

**Table 1:** Demographic data of 234 primary healthcare physicians in Jazan, Saudi Arabia.

Variables:	
Age: median [minimum–maximum]^∗^	38 yr [25–65]
Gender: n [%]	
Male	113 [48.3%]
Females	121 [51.7%]
Nationality: n [%]	
Sudanese	129 [55.1%]
Saudis	28 [12%]
Egyptian	26 [11.1%]
Pakistani	20 [8.5%]
Indian	9 [3.8%]
Syrian	8 [3.4%]
Cuban	8 [3.4%]
Nigerian	5 [2.1]
Palestinian	1 [0.4%]
Specialty: n [%]	
General practitioners	168 [71.8%]
Family medicine	66 [28.2%]
Highest academic degree: n [%]	
MBBS	159 [67.9%]
High diploma	34 [14.5%]
Masters	27 [11.5%]
Board	10 [4.3%]
Fellowship	4 [1.7%]
Number of years of practice: median [minimum–maximum]	10 yr [Less than a year – 43]
Average number of patients seen on daily basis^∗^: mean [SD]	40 patients [17.9]

### 3.2 Knowledge concerning prevention of obesity and hypercholesterolemia

Knowledge of the physicians concerning the prevention of obesity and hypercholesterolemia is summarised in Table 2. The majority of the physicians understand that physical activity can reduce the risk of atherosclerotic cardiovascular diseases, even without weight loss (97%). Similarly, the majority of the physicians understand that psychological interventions should be a part of any weight management programme (90%). However, knowledge of the physicians about the eat-well plate recommendations was very low, with none of the physicians reporting the recommended daily portions of fruits and vegetables. Additionally, the minority of the physicians were knowledgeable about BMI cutoff points for considering the initiation of pharmacotherapy or bariatric surgery. Finally, <20% of the physicians knew the cutoff points for considering central obesity among male and female individuals.

**Table 2:** Knowledge of 234 primary healthcare physicians in Jazan, Saudi Arabia about the prevention of obesity and hypercholesterolemia.

Knowledge items:	Frequency of correct answers [percentages]
Eat-well plate recommendations concerning bread, rice, potatoes, pasta, and other starchy food products.	9 [4%]
Eat-well plate recommendations concerning dairy and meat products	14 [6%]
Eat-well plate recommendations concerning fruits and vegetables consumption	0 [0%]
Eat-well plate recommendations concerning food items rich with high fat/sugar	131 [56%]
Recommended minimum required number of minutes of moderate to vigorous activities for the prevention of obesity among children	48 [21%]
Recommended maximum number of hours to be spent on sedentary activities such as TV watching or mobile use on a daily basis among children	101 [43%]
Effect of physical activity without weight loss on reducing risk cardiovascular diseases and type 2 diabetes risk	125 [53%]
Recommendation concerning weight loss among children and adolescents	173 [74%]
BMI level where pharmacotherapy is recommended to treat obesity among adolescents	33 [14%]
Cutoff point of waist circumference to consider central obesity among males	44 [19%]
Cutoff point of waist circumference to consider central obesity among females	37 [16%]
Psychological interventions within weight management program	210 [90%]
Level of BMI indicating need for bariatric surgery among adults having poorly control diabetes mellitus and risk of cardiovascular diseases	38 [16%]
Level of BMI indicating need for bariatric surgery among adults not suffering from any comorbidities	153 [65%]
Recommendation concerning the measurement of LDL-C among adult aged 20 years or older who are not at risk of atherosclerotic cardiovascular disease	87 [37%]
Effect of physical activity on the lifetime risk of atherosclerotic cardiovascular disease	228 [97%]
Recommendation concerning the prescription of statin for individuals under 20 as measure to prevent cardiovascular diseases	35 [15%]
Recommendation concerning prescribing statin for adults aged 21 years with LDL-C ≥4.1 mmol/L and a family history of premature atherosclerotic cardiovascular disease	191 [82%]
Median level of knowledge: 7 [IQR: 6–8]. Minimum score: 2. Highest score: 14	

### 3.3 Practice concerning prevention of obesity and hypercholesterolemia

Table 3 describes the practice of physicians concerning the prevention of obesity and hypercholesterolemia. The majority of physicians reported reading clinical guidelines pertaining to the prevention of diseases. However, only 21% of the physicians reported adherence concerning screening for obesity and 42% reported adherence to the guidelines concerning screening for hypercholesterolemia. The majority of physicians reported providing lifestyle interventions to patients visiting their clinics to reduce obesity and hypercholesterolemia. However, only 9% of the physicians were adherent to the guidelines concerning reducing the risk of obesity and only 13% reported adherence to the guidelines related to the reduction of hypercholesterolemia risk. Most of the physicians provided general advice concerning eating well or more engagement in physical activity but without describing specific goals to be achieved by their patients.

**Table 3:** Practice of 234 primary healthcare physicians in Jazan region concerning prevention of obesity and hypercholesterolemia.

Practice Statement	Frequency [percentage]
Physicians who reported reading guidelines as a source of information about the prevention of obesity and hypercholesterolemia	225 [96%]
Physicians adherent to the guidelines concerning criteria for screening for obesity	50 [21%]
Physicians adherent to guidelines concerning criteria for screening for hypercholesterolemia	99 [42%]
Physicians who reported provision of lifestyle interventions for the patients	225 [96%]
Physicians adherent to the guidelines concerning lifestyle interventions to reduce risk of obesity	20 [9%]
Physicians adherent to the guidelines concerning lifestyle interventions to reduce the risk of hypercholesterolemia	30 [13%]

### 3.4 Associations between knowledge and practice level and measured physicians characteristics

Associations between physician demographics and level of knowledge or adherence concerning the prevention of obesity and hypercholesterolemia are shown in Table 4. None of the tested associations were statistically significant. This can be partially explained by the fact that level of knowledge and practice adherence were generally low in the recruited sample resulting with no detected statistically significant difference concerning level of knowledge and adherence between the comparison groups.

**Table 4:** Factors associated with level of knowledge and adherence to guidelines of 234 primary healthcare physicians in Jazan region concerning prevention of obesity and dyslipidemia.

Variables	Odds of higher knowledge [95% CI]	*P* value	Odds of practice adherence [95% CI]	*P* value
Age:				
<38 yr	1.57 [0.91–2.72]	.11	0.79 [0.47–1.34]	.39
38 yr or older^∗^				
Gender: n [%]				
Male^∗^	0.95 [0.55–1.63]	.87	0.84 [0.49–1.42]	.51
Female				
Nationality according to language: n [%]				
Native Arabic speakers	1.50 [0.75–2.96]	.91	1.06 [0.53–2.09]	.86
Non-native Arabic speakers^∗^				
Specialty: n [%]				
General practitioners	1.18 [0.65–2.14]	.56	1.19 [0.67–2.13]	.54
Family medicine^∗^				
Highest academic degree				
MBBS^∗^	0.97 [0.54–1.73]	.93	1.22 [0.69–2.16]	.47
Postgraduate education				
Number of years of practice				
<10 yr	1.60 [0.933–2.76]	.08	0.86 [0.51–1.46]	.59
10 yr or more^∗^				
Average number of patients seen on daily basis				
<40 patients	1.05 [0.61–1.81]	.83	1.08 [0.63–1.83]	.77
40 patients or more^∗^				

## 4 Discussion

This study aimed to measure knowledge and adherence of PHC physicians in Jazan region concerning the prevention of obesity and hypercholesterolemia, where multiple deficiencies in knowledge and limited adherence were detected. The physicians’ knowledge about eat-well plate guidelines was limited. Additionally, physician knowledge about BMI cutoff points to consider pharmacotherapy or bariatric surgery to prevent complications of obesity was low, indicating the potential limited ability of PHC physicians in deciding whether patients require referral to specialised centres for the management of obesity. Furthermore, the minority of physicians reported adherence to screening and lifestyle modification guidelines to reduce the risk of obesity and hypercholesterolemia, indicating low engagement of the study's sample of physicians in the prevention of disease.

The findings of this study can be compared to other similar local investigations. A study was conducted by Sebiany involving 130 PHCs in Dammam, in Eastern province in Saudi Arabia; the study reported that two thirds of the respondents considered themselves contributors in the prevention and management of obesity. However, only one third believed that they were well-trained to treat obesity. Lack of training, limited administrative support and time constraints were reported as factors influencing the management of obesity in their clinics.^[14]^ These findings are similar to ours, where the majority of physicians declared that they were screening for obesity and hypercholesterolemia. However, only the minority showed adherence to prevention guidelines.

A recent study was conducted by Al-Noor et al^[15]^ in Riyadh city and involved 199 physicians, where knowledge, attitude, feelings and practice about obesity were measured. The majority of the physicians in their sample reported the provision of dietary advice and physical activity advice for their patients. However, it was not clear whether the provided advice was adherent to the available clinical guidelines concerning obesity management. Additionally, less than half of their sample of physicians (48%) reported recording the body weight of patients visiting their clinics.^[15]^

Studies measuring the level of knowledge of PHCs about the prevention of hypercholesterolemia in Saudi Arabia are lacking. However, the limited knowledge of physicians in our sample concerning the prevention of hypercholesterolemia can be compared to other investigations assessing the management of familial hypercholesterolemia. In a study measuring the knowledge of 225 family physicians in Riyadh, Saudi Arabia, about familial hypercholesterolemia, it was reported that nearly half of the physician had poor knowledge of the disease. However, 65% of the physicians screened the relatives of diagnosed patients for the disease.^[16]^ A similar study conducted in Riyadh and targeting physicians working at tertiary healthcare settings reported a deficit in knowledge and practice concerning screening for and the management of familial hypercholesterolaemia.^[17]^

PHCs could be involved in community-based interventions to reduce obesity. However, there is currently no screening policy for obesity and hypercholesterolemia in Saudi Arabia.^[18]^ In a review by Alnaami concerning the prevention and control of obesity, a comprehensive multilevel approach was described where different settings have been recommended to be involved in obesity prevention programmes including PHCs.^[19]^

Several studies elaborated on the importance of involving general practitioners and PHCs in overall weight management.^[20–22]^ A systematic review assessed the effectiveness of physical activity promotion in primary care settings, where 15 interventional trials were identified. It was revealed that physical activity promotion in primary care settings can significantly increase patients’ level of physical activity at 12 months of the interventions.^[23]^ However, in a French study involving 600 general practitioners, 57% of the physicians were pessimistic about their patients’ ability to lose weight, indicating a lack of physician motivation and a feeling of ineffectiveness about their role in the prevention and control of obesity.^[21]^ Nonetheless, the constraints that primary healthcare settings can impose on the overall involvement of physicians in the prevention and management of obesity must be acknowledged.^[24]^

The current investigation was able to identify areas of weaknesses with regard to knowledge and reported adherence of PHC physicians in Jazan, Saudi Arabia concerning prevention of obesity and hypercholesterolemia. Although the findings of the logistic regression did not reveal any associations between the measured demographics and clinical practice characteristics and knowledge and reported adherence of the recruited physicians, it must be noted that the limited knowledge or incompliance of the physicians to the guidelines can be partially explained by the possible gap between the developed clinical guidelines and the real-world practice. This notion has been raised by several reports presenting a debate calling for a need to balance the strict designs of clinical trials used to derive practice guidelines with the real-world data, especially with the availability of electronic health records.^[25–27]^ For example, in a large scale Chinese study involving more than 85,000 inpatients and outpatients with diabetes, it was concluded that only 17.4% of the inpatients and 7.2% of the outpatients met the inclusion criteria for a pragmatic randomized trial suggesting a difference of patients characteristics between real-world hospital settings to pragmatic clinical trials.^[28]^

This study has multiple strength and weaknesses. Its strengths are mainly related to targeting physicians working at PHCs in urban and rural areas in Jazan region via interviews and utilising open-ended questions to measure the level of knowledge or practice adherence. The weaknesses can be related to measurements of reported adherence rather than observing the actual practice of physicians. However, the study was able to identify several areas of deficiencies in knowledge or practice, indicating the limited role of PHC physicians in the prevention of obesity and hypercholesterolemia in Jazan region.

In conclusion, the evidence generated from our investigation is consistent with similar studies conducted locally or internationally, indicating a limited role of PHC physicians in the prevention of obesity or hypercholesterolemia. Knowledge of the recruited PHCs physicians was limited with regard to obesity and hypercholesterolemia prevention. Similarly, the adherence of physicians to screening guidelines and lifestyle counselling of their patients was limited. This reflects a need to develop training programs targeting PHC physicians to increase their knowledge about the guidelines and to increase their adherence. Additionally, more emphasis should be made to ensure provision of sufficient staffing and infrastructure to PHCs in the region to enable multidisciplinary collaboration between healthcare professionals including physicians, nutritionists and health educationists.

## Acknowledgments

We wish to acknowledge the valuable contribution of Dr Abdul-Rahaman Salim, Consultant of Family Medicine at Directory of Health Affairs in Jazan and his review the developed study instrument.

## Author contributions

IG was responsible for the study concept and design, development and testing of the data collection tool, responsible for data collection supervision, data entry, and analysis and prepared the final draft of the manuscript.

**Conceptualization:** Ibrahim Gosadi.

**Data curation:** Ibrahim Gosadi.

**Formal analysis:** Ibrahim Gosadi.

**Investigation:** Ibrahim Gosadi.

**Methodology:** Ibrahim Gosadi.

**Project administration:** Ibrahim Gosadi.

**Writing – original draft:** Ibrahim Gosadi.

**Writing – review & editing:** Ibrahim Gosadi.
